# Supplementary material for: HIVprotI: an integrated web based platform for prediction and design of HIV proteins inhibitors
Source: J Cheminform. 2018 Mar 9;10:12. doi: 10.1186/s13321-018-0266-y (PMC5845081; doi:10.1186/s13321-018-0266-y)
Supplement: Supplementary file 2 — Additional file 2. Source code of HIVProtI web server. [file 13321_2018_266_MOESM2_ESM.zip › HIVprotI_Source-code/hivprotI/weka-3-6-6/doc/weka/datagenerators/clusterers/SubspaceCluster.html]

SubspaceCluster


---


|  |  |  |
| --- | --- | --- |
| **Overview** \| **Package** \| **Class** \| **Tree** \| **Deprecated** \| **Index** \| **Help** \| **Weka's home** | |  |
| **PREV CLASS**   **NEXT CLASS** | **FRAMES**    **NO FRAMES**     **All Classes** |  |
| SUMMARY: NESTED \| FIELD \| CONSTR \| METHOD | DETAIL: FIELD \| CONSTR \| METHOD |  |


---


## weka.datagenerators.clusterers Class SubspaceCluster

```
java.lang.Object
  weka.datagenerators.DataGenerator
      weka.datagenerators.ClusterGenerator
          weka.datagenerators.clusterers.SubspaceCluster
```

**All Implemented Interfaces:**: java.io.Serializable, OptionHandler, Randomizable, RevisionHandler

---

``` public class SubspaceCluster extends ClusterGenerator ```

A data generator that produces data points in hyperrectangular subspace clusters.


Valid options are:

```
 -h
  Prints this help.
```

```
 -o <file>
  The name of the output file, otherwise the generated data is
  printed to stdout.
```

```
 -r <name>
  The name of the relation.
```

```
 -d
  Whether to print debug informations.
```

```
 -S
  The seed for random function (default 1)
```

```
 -a <num>
  The number of attributes (default 1).
```

```
 -c
  Class Flag, if set, the cluster is listed in extra attribute.
```

```
 -b <range>
  The indices for boolean attributes.
```

```
 -m <range>
  The indices for nominal attributes.
```

```
 -P <num>
  The noise rate in percent (default 0.0).
  Can be between 0% and 30%. (Remark: The original 
  algorithm only allows noise up to 10%.)
```

```
 -C <cluster-definition>
  A cluster definition of class 'SubspaceClusterDefinition'
  (definition needs to be quoted to be recognized as 
  a single argument).
```

```
 Options specific to weka.datagenerators.clusterers.SubspaceClusterDefinition:
```

```
 -A <range>
  Generates randomly distributed instances in the cluster.
```

```
 -U <range>
  Generates uniformly distributed instances in the cluster.
```

```
 -G <range>
  Generates gaussian distributed instances in the cluster.
```

```
 -D <num>,<num>
  The attribute min/max (-A and -U) or mean/stddev (-G) for
  the cluster.
```

```
 -N <num>..<num>
  The range of number of instances per cluster (default 1..50).
```

```
 -I
  Uses integer instead of continuous values (default continuous).
```

**Version:**
:   $Revision: 1.5 $

**Author:**
:   Gabi Schmidberger (gabi@cs.waikato.ac.nz), FracPete (fracpete at waikato dot ac dot nz)

**See Also:**: Serialized Form

---

| **Field Summary** | |
| --- | --- |
| `static int` | `CONTINUOUS`             cluster subtype: continuous |
| `static int` | `GAUSSIAN`             cluster type: gaussian |
| `static int` | `INTEGER`             cluster subtype: integer |
| `static Tag[]` | `TAGS_CLUSTERSUBTYPE`             the tags for the cluster types |
| `static Tag[]` | `TAGS_CLUSTERTYPE`             the tags for the cluster types |
| `static int` | `TOTAL_UNIFORM`             cluster type: total uniform |
| `static int` | `UNIFORM_RANDOM`             cluster type: uniform/random |


**Constructor Summary**

`SubspaceCluster()`
  
          initializes the generator, sets the number of clusters to 0, since user
has to specify them explicitly


| **Method Summary** | |
| --- | --- |
| `java.lang.String` | `clusterDefinitionsTipText()`             Returns the tip text for this property |
| `Instances` | `defineDataFormat()`             Initializes the format for the dataset produced. |
| `Instance` | `generateExample()`             Generate an example of the dataset. |
| `Instances` | `generateExamples()`             Generate all examples of the dataset. |
| `java.lang.String` | `generateFinished()`             Compiles documentation about the data generation after the generation process |
| `java.lang.String` | `generateStart()`             Compiles documentation about the data generation before the generation process |
| `ClusterDefinition[]` | `getClusterDefinitions()`             returns the currently set clusters |
| `double` | `getNoiseRate()`             Gets the percentage of noise set. |
| `int[]` | `getNumValues()`             returns array that stores the number of values for a nominal attribute. |
| `java.lang.String[]` | `getOptions()`             Gets the current settings of the datagenerator. |
| `java.lang.String` | `getRevision()`             Returns the revision string. |
| `boolean` | `getSingleModeFlag()`             Gets the single mode flag. |
| `java.lang.String` | `globalInfo()`             Returns a string describing this data generator. |
| `boolean` | `isBoolean(int index)`             Returns true if attribute is boolean |
| `boolean` | `isNominal(int index)`             Returns true if attribute is nominal |
| `java.util.Enumeration` | `listOptions()`             Returns an enumeration describing the available options. |
| `static void` | `main(java.lang.String[] args)`             Main method for testing this class. |
| `java.lang.String` | `noiseRateTipText()`             Returns the tip text for this property |
| `java.lang.String` | `numAttributesTipText()`             Returns the tip text for this property |
| `void` | `setClusterDefinitions(ClusterDefinition[] value)`             sets the clusters to use |
| `void` | `setNoiseRate(double newNoiseRate)`             Sets the percentage of noise set. |
| `void` | `setNumAttributes(int numAttributes)`             Sets the number of attributes the dataset should have. |
| `void` | `setOptions(java.lang.String[] options)`             Parses a list of options for this object. |

**Methods inherited from class weka.datagenerators.ClusterGenerator**

`booleanColsTipText, classFlagTipText, getBooleanCols, getClassFlag, getNominalCols, getNumAttributes, nominalColsTipText, setBooleanCols, setBooleanIndices, setClassFlag, setNominalCols, setNominalIndices`

**Methods inherited from class weka.datagenerators.DataGenerator**

`debugTipText, defaultOutput, formatTipText, getDatasetFormat, getDebug, getNumExamplesAct, getOutput, getRandom, getRelationName, getSeed, makeData, outputTipText, randomTipText, relationNameTipText, seedTipText, setDatasetFormat, setDebug, setOutput, setRandom, setRelationName, setSeed`

**Methods inherited from class java.lang.Object**

`equals, getClass, hashCode, notify, notifyAll, toString, wait, wait, wait`

**Field Detail**

### UNIFORM\_RANDOM

```
public static final int UNIFORM_RANDOM
```

:   cluster type: uniform/random

    **See Also:**: Constant Field Values

---


### TOTAL\_UNIFORM

```
public static final int TOTAL_UNIFORM
```

:   cluster type: total uniform

    **See Also:**: Constant Field Values

---


### GAUSSIAN

```
public static final int GAUSSIAN
```

:   cluster type: gaussian

    **See Also:**: Constant Field Values

---


### TAGS\_CLUSTERTYPE

```
public static final Tag[] TAGS_CLUSTERTYPE
```

:   the tags for the cluster types

---


### CONTINUOUS

```
public static final int CONTINUOUS
```

:   cluster subtype: continuous

    **See Also:**: Constant Field Values

---


### INTEGER

```
public static final int INTEGER
```

:   cluster subtype: integer

    **See Also:**: Constant Field Values

---


### TAGS\_CLUSTERSUBTYPE

```
public static final Tag[] TAGS_CLUSTERSUBTYPE
```

:   the tags for the cluster types


**Constructor Detail**

### SubspaceCluster

```
public SubspaceCluster()
```

:   initializes the generator, sets the number of clusters to 0, since user
    has to specify them explicitly


**Method Detail**

### globalInfo

```
public java.lang.String globalInfo()
```

:   Returns a string describing this data generator.

    :   **Returns:**: a description of the data generator suitable for displaying in the explorer/experimenter gui

---


### listOptions

```
public java.util.Enumeration listOptions()
```

:   Returns an enumeration describing the available options.

    :   **Specified by:**: `listOptions` in interface `OptionHandler` **Overrides:**: `listOptions` in class `ClusterGenerator`
    :   **Returns:**: an enumeration of all the available options

---


### setOptions

```
public void setOptions(java.lang.String[] options)
                throws java.lang.Exception
```

:   Parses a list of options for this object.


    Valid options are:

    ```
     -h
      Prints this help.
    ```

    ```
     -o <file>
      The name of the output file, otherwise the generated data is
      printed to stdout.
    ```

    ```
     -r <name>
      The name of the relation.
    ```

    ```
     -d
      Whether to print debug informations.
    ```

    ```
     -S
      The seed for random function (default 1)
    ```

    ```
     -a <num>
      The number of attributes (default 1).
    ```

    ```
     -c
      Class Flag, if set, the cluster is listed in extra attribute.
    ```

    ```
     -b <range>
      The indices for boolean attributes.
    ```

    ```
     -m <range>
      The indices for nominal attributes.
    ```

    ```
     -P <num>
      The noise rate in percent (default 0.0).
      Can be between 0% and 30%. (Remark: The original 
      algorithm only allows noise up to 10%.)
    ```

    ```
     -C <cluster-definition>
      A cluster definition of class 'SubspaceClusterDefinition'
      (definition needs to be quoted to be recognized as 
      a single argument).
    ```

    ```
     Options specific to weka.datagenerators.clusterers.SubspaceClusterDefinition:
    ```

    ```
     -A <range>
      Generates randomly distributed instances in the cluster.
    ```

    ```
     -U <range>
      Generates uniformly distributed instances in the cluster.
    ```

    ```
     -G <range>
      Generates gaussian distributed instances in the cluster.
    ```

    ```
     -D <num>,<num>
      The attribute min/max (-A and -U) or mean/stddev (-G) for
      the cluster.
    ```

    ```
     -N <num>..<num>
      The range of number of instances per cluster (default 1..50).
    ```

    ```
     -I
      Uses integer instead of continuous values (default continuous).
    ```

    :   **Specified by:**: `setOptions` in interface `OptionHandler` **Overrides:**: `setOptions` in class `ClusterGenerator`
    :   **Parameters:**: `options` - the list of options as an array of strings **Throws:**: `java.lang.Exception` - if an option is not supported

---


### getOptions

```
public java.lang.String[] getOptions()
```

:   Gets the current settings of the datagenerator.

    :   **Specified by:**: `getOptions` in interface `OptionHandler` **Overrides:**: `getOptions` in class `ClusterGenerator`
    :   **Returns:**: an array of strings suitable for passing to setOptions **See Also:**: `DataGenerator.removeBlacklist(String[])`

---


### setNumAttributes

```
public void setNumAttributes(int numAttributes)
```

:   Sets the number of attributes the dataset should have.

    :   **Overrides:**: `setNumAttributes` in class `ClusterGenerator`
    :   **Parameters:**: `numAttributes` - the new number of attributes

---


### numAttributesTipText

```
public java.lang.String numAttributesTipText()
```

:   Returns the tip text for this property

    :   **Overrides:**: `numAttributesTipText` in class `ClusterGenerator`
    :   **Returns:**: tip text for this property suitable for displaying in the explorer/experimenter gui

---


### getNoiseRate

```
public double getNoiseRate()
```

:   Gets the percentage of noise set.

    :   **Returns:**: the percentage of noise set

---


### setNoiseRate

```
public void setNoiseRate(double newNoiseRate)
```

:   Sets the percentage of noise set.

    :   **Parameters:**: `newNoiseRate` - new percentage of noise

---


### noiseRateTipText

```
public java.lang.String noiseRateTipText()
```

:   Returns the tip text for this property

    :   **Returns:**: tip text for this property suitable for displaying in the explorer/experimenter gui

---


### getClusterDefinitions

```
public ClusterDefinition[] getClusterDefinitions()
```

:   returns the currently set clusters

    :   **Returns:**: the currently set clusters

---


### setClusterDefinitions

```
public void setClusterDefinitions(ClusterDefinition[] value)
                           throws java.lang.Exception
```

:   sets the clusters to use

    :   **Parameters:**: `value` - the clusters do use **Throws:**: `java.lang.Exception` - if clusters are not the correct class

---


### clusterDefinitionsTipText

```
public java.lang.String clusterDefinitionsTipText()
```

:   Returns the tip text for this property

    :   **Returns:**: tip text for this property suitable for displaying in the explorer/experimenter gui

---


### getSingleModeFlag

```
public boolean getSingleModeFlag()
```

:   Gets the single mode flag.

    :   **Specified by:**: `getSingleModeFlag` in class `DataGenerator`
    :   **Returns:**: true if methode generateExample can be used.

---


### defineDataFormat

```
public Instances defineDataFormat()
                           throws java.lang.Exception
```

:   Initializes the format for the dataset produced.

    :   **Overrides:**: `defineDataFormat` in class `DataGenerator`
    :   **Returns:**: the output data format **Throws:**: `java.lang.Exception` - data format could not be defined **See Also:**: `DataGenerator.defaultRelationName()`

---


### isBoolean

```
public boolean isBoolean(int index)
```

:   Returns true if attribute is boolean

    :   **Parameters:**: `index` - of the attribute **Returns:**: true if the attribute is boolean

---


### isNominal

```
public boolean isNominal(int index)
```

:   Returns true if attribute is nominal

    :   **Parameters:**: `index` - of the attribute **Returns:**: true if the attribute is nominal

---


### getNumValues

```
public int[] getNumValues()
```

:   returns array that stores the number of values for a nominal attribute.

    :   **Returns:**: the array that stores the number of values for a nominal attribute

---


### generateExample

```
public Instance generateExample()
                         throws java.lang.Exception
```

:   Generate an example of the dataset.

    :   **Specified by:**: `generateExample` in class `DataGenerator`
    :   **Returns:**: the instance generated **Throws:**: `java.lang.Exception` - if format not defined or generating

---


### generateExamples

```
public Instances generateExamples()
                           throws java.lang.Exception
```

:   Generate all examples of the dataset.

    :   **Specified by:**: `generateExamples` in class `DataGenerator`
    :   **Returns:**: the instance generated **Throws:**: `java.lang.Exception` - if format not defined

---


### generateFinished

```
public java.lang.String generateFinished()
                                  throws java.lang.Exception
```

:   Compiles documentation about the data generation after
    the generation process

    :   **Specified by:**: `generateFinished` in class `DataGenerator`
    :   **Returns:**: string with additional information about generated dataset **Throws:**: `java.lang.Exception` - no input structure has been defined

---


### generateStart

```
public java.lang.String generateStart()
```

:   Compiles documentation about the data generation before
    the generation process

    :   **Specified by:**: `generateStart` in class `DataGenerator`
    :   **Returns:**: string with additional information

---


### getRevision

```
public java.lang.String getRevision()
```

:   Returns the revision string.

    :   **Returns:**: the revision

---


### main

```
public static void main(java.lang.String[] args)
```

:   Main method for testing this class.

    :   **Parameters:**: `args` - should contain arguments for the data producer:


---


|  |  |  |
| --- | --- | --- |
| **Overview** \| **Package** \| **Class** \| **Tree** \| **Deprecated** \| **Index** \| **Help** \| **Weka's home** | |  |
| **PREV CLASS**   **NEXT CLASS** | **FRAMES**    **NO FRAMES**     **All Classes** |  |
| SUMMARY: NESTED \| FIELD \| CONSTR \| METHOD | DETAIL: FIELD \| CONSTR \| METHOD |  |


---
